# Supplementary figures and images for: Towards the sustainable elimination of gambiense human African trypanosomiasis in Côte d’Ivoire using an integrated approach
Source: PLoS Negl Trop Dis. 2023 Jul 31;17(7):e0011514. doi: 10.1371/journal.pntd.0011514 (PMC10443840; doi:10.1371/journal.pntd.0011514)

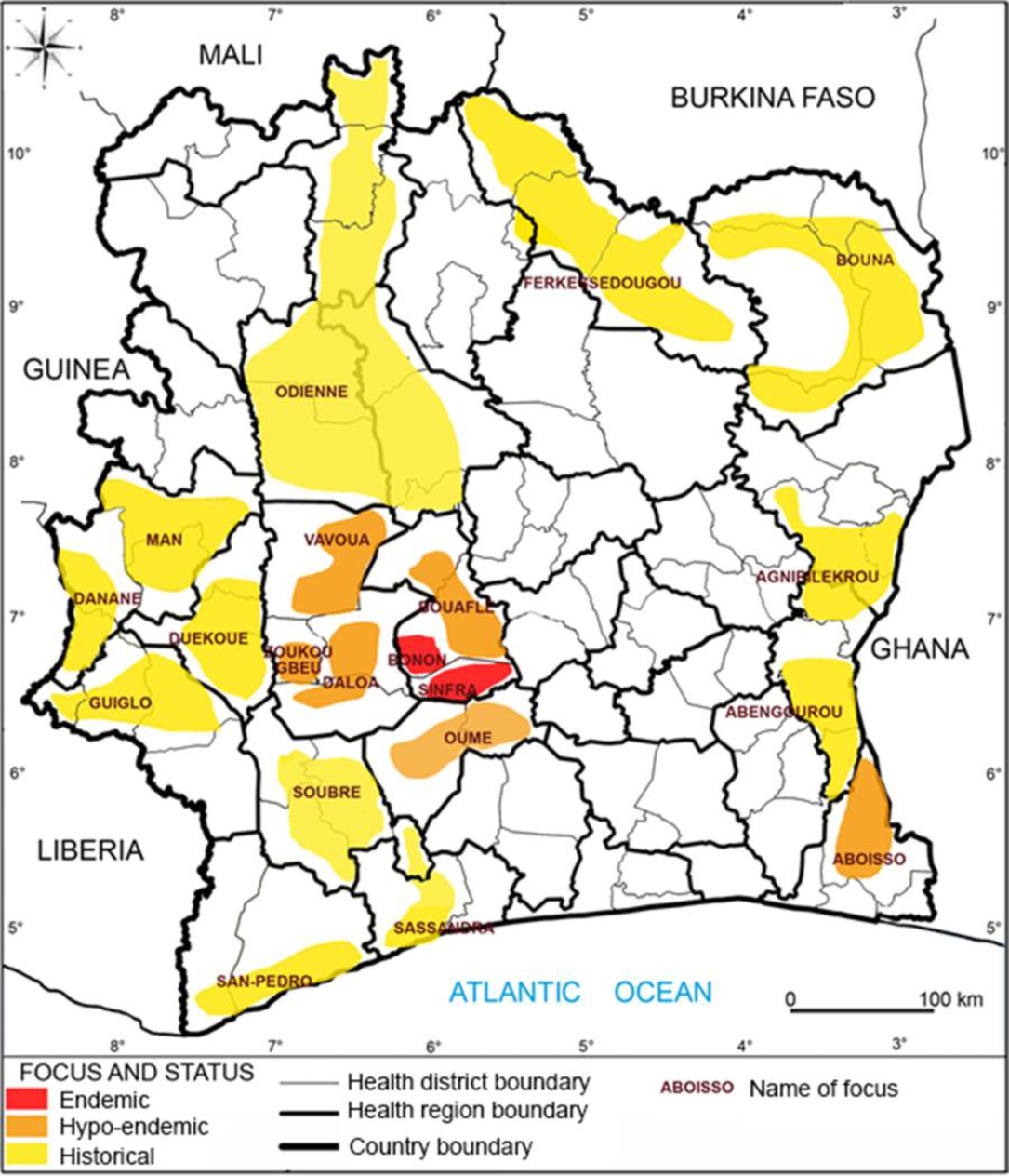

Supplement: S1 Fig — This figure was created by the mapping service of our team based at Institut Pierre Richet (Bouaké, Côte d’Ivoire). All the base layers regarding administrative data are available in: https://data.humdata.org/dataset/cote-d-ivoire-roads, https://data.humdata.org/dataset/cote-d-ivoire-settlements and https://data.humdata.org/dataset/cod-ab-civ (TIF) [file pntd.0011514.s005.tif]
